# Subnational regional inequality in access to improved drinking water and sanitation in Indonesia: results from the 2015 Indonesian National Socioeconomic Survey (SUSENAS)

**DOI:** 10.1080/16549716.2018.1496972

**Published:** 2018-08-01

**Authors:** Tin Afifah, Mariet Tetty Nuryetty, Cahyorini, Dede Anwar Musadad, Anne Schlotheuber, Nicole Bergen, Richard Johnston

**Affiliations:** a National Institute of Health Research and Development, Ministry of Health, Jakarta, Indonesia; b Badan Pusat Statistik, BPS – Statistics Indonesia, Jakarta, Indonesia; c Department of Information, Evidence and Research, World Health Organization, Geneva, Switzerland; d Faculty of Health Sciences, University of Ottawa, Ottawa, Canada; e Department of Public Health, Environmental and Social Determinants of Health, World Health Organization, Geneva, Switzerland

**Keywords:** Monitoring Health Inequality in Indonesia, Indonesia, water, sanitation, health inequality, health equity

## Abstract

**Background**: Universal and equitable access to safe and affordable drinking water and adequate sanitation and hygiene in Indonesia are vital to ensure healthy lives and promote well-being for all at all ages.

**Objectives**: To quantify subnational regional inequality in access to improved drinking water and sanitation in Indonesia.

**Methods**: Data about access to improved drinking water and sanitation were derived from the 2015 Indonesian National Socioeconomic Survey (SUSENAS) and disaggregated by 510 districts across the 34 provinces of Indonesia. Two summary measures of inequality, mean difference from mean and weighted index of disparity, were calculated to quantify within-province absolute and relative inequality, respectively.

**Results**: While the majority of Indonesian households had access to improved drinking water (71.0%) and sanitation (62.1%), there were large variations between and within provinces. Access to improved drinking water ranged from 93.4% in DKI Jakarta to 41.1% in Bengkulu, and access to improved sanitation ranged from 89.3% in Jakarta to 23.9% in East Nusa Tenggara. Provinces with similar numbers of districts and similar overall averages showed variable levels of absolute and/or relative inequality. Certain districts reported very low levels of access to improved drinking water and/or sanitation.

**Conclusions**: There are inequalities in access to improved drinking water and sanitation by subnational region in Indonesia. Monitoring within-country inequality in these indicators serves to identify underserved areas, and is useful for developing approaches to improve inequalities in access that can help Indonesia make progress towards the 2030 Agenda for Sustainable Development.

## Background

In Indonesia, water and sanitation remains a pressing public health issue, with broad implications for health and development. Universal and equitable access to safe and affordable drinking water and sanitation are fundamental to population health and well-being. Having access to improved drinking water and sanitation is directly related to the prevention of disease and death from diarrhoeal disease, trachoma and intestinal helminths (Ascaris, Trichuris, hookworm); it also helps to mitigate risks associated with malnutrition (i.e. resulting from the inability to derive nutritional value from food) and the underlying determinants of malnutrition [1–5]. Improvements in drinking water and sanitation convey significant economic returns through improved health; these returns outweigh the cost of supplying water and sanitation services [6].

Increasing access to improved drinking water and sanitation was a priority in the Millennium Development Goals (MDGs) with target 7c specifying a halving, by 2015, of the proportion of people without sustainable access to safe drinking water and basic sanitation [7]. Indonesia was successful in meeting the MDG target for improved drinking water, with 90% of the population using improved drinking water sources in 2015 (an increase of 15 percentage points since 2000) [8]. The country made ‘good progress’ towards achieving the MDG target for improved sanitation, with national levels of 68% in 2015 (though less than the average of 77% across countries of the Eastern and South-Eastern Asia Region) [8].

Indonesia, despite realizing considerable national progress during the MDG era (2000–2015), has reported geographical variations in access to improved drinking water and sanitation [8–10]. Rural–urban gaps were notable, with lower access in rural than in urban areas [8,9]. The island of Java, location of capital city Jakarta and a top economic performer in the country, fared better than other islands in Indonesia [10]. The eastern provinces, especially Papua and West Papua, tended to fare much worse than other provinces [9,10].

The equity-oriented Sustainable Development Goals (SDG) targets for ‘universal and equitable access to safe and affordable drinking water for all’, and ‘adequate and equitable sanitation and hygiene for all’ necessitate regular within-country inequality monitoring [11]. While previous studies have explored inequalities between urban and rural areas, between islands and between different provinces, to our knowledge no previous study has investigated within-province inequalities in access to improved drinking water and sanitation in Indonesia (i.e. at the district level or lower). In this study, we draw from the National Socioeconomic Survey (SUSENAS), which helps to provide an overview of Indonesia’s state of inequality in water and sanitation indicators at the district level. Indeed, within low- and middle-income country settings it is rare to have a household survey with a large sample size and representativeness at the district level (we are aware of only two other countries that have this type of data available: the 2015 National Family Health Surveys in India and the 2000 Demographic and Health Surveys in Iran). This study aimed to quantify inequality in the use of improved drinking water sources and sanitation facilities between and within provinces using district-level data in Indonesia. The study conveys a novel approach to quantify district-level inequality in water and sanitation, and additionally expounds upon the challenges faced by the adoption of a new global SDG indicator and its applicability within the national context of Indonesia.

SDGs, notably in Goal 6: to ensure availability and sustainable management of water and sanitation for all [12]. Water and sanitation are also related to several other SDGs, in particular those addressing health, poverty, nutrition, economic growth and work [12]. Like the MDGs, the SDGs reflect a conceptual approach that assesses progress through quantitative indicators, which are applied at the national level. In the case of SDG targets 6.1 and 6.2 on drinking water and sanitation, respectively, new indicators have been developed for the SDGs which address the type of infrastructure used and also the quality of the service delivered. ‘Safely managed drinking water and sanitation services’, the indicators for these targets, build on the MDG indicators of ‘use of improved facilities’ but also include the accessibility, availability and quality of water services, and the ways in which excreta are treated and disposed of, both in sewer and on-site sanitation systems [8].

Due to the ambitious nature of the water and sanitation SDG indicators, the levels of coverage reflected by these indicators will be substantially lower than the level of coverage reflected in the MDG indicators, and many countries are unlikely to achieve 100% coverage by 2030 [13]. However, the 2030 Agenda for Sustainable Development calls upon countries to set ambitious but achievable national targets, rather than apply the same target of 100% coverage by 2030 in all settings [11].

Another implication of the new SDG indicators is that they require more data than the MDG indicators, and monitoring systems need to be adapted and updated in order to report against the new indicators. In response to the data requirements for SDG monitoring, new questions and modules have been developed for inclusion in household surveys [14]; however, information on the quality of drinking water and sanitation services can more appropriately be obtained from government authorities that have regulatory oversight of service providers, and a mandate to monitor and value water resources and services [15].

## Methods

The SUSENAS is a multi-purpose household survey conducted twice a year (in March and September) in Indonesia, covering 300,000 households in all districts; 95% of districts have a sample size of at least 360 households [16]. The SUSENAS has been conducted since 1979. The sample design of the SUSENAS, which uses probability samples, allows for estimation of district-level coverage; thus the SUSENAS is an appropriate data source for monitoring inequality at both district and province levels. Briefly, the samples were selected by a three-step sampling approach: step 1 entailed selecting 25% of the total census blocks by applying probability proportional to size; in step 2, a number of census blocks were selected by applying systematic sampling in each urban/rural strata in each district, by wealth strata; and in step 3, 10 households were selected by applying systematic sampling with implicit stratification of the highest level of education attained by the household head. For this study, data were derived from the March 2015 SUSENAS, which are representative at the district level. Detailed information about the survey and the sampling design and census block allocation are available at: http://microdata.bps.go.id/mikrodata/index.php/catalog/653/related_materials (in Bahasa).

The SUSENAS collects the data required to measure access to improved drinking water and sanitation, but not safely managed services: in particular, data on drinking water quality and excreta management are not captured in the survey. Our analysis is therefore restricted to the population using improved water and sanitation facilities, using national definitions. The global MDG indicator of ‘use of an improved drinking water source’ considers improved drinking water sources to include piped water and public tap/standpipe as well as borehole/tube well, protected dug well, protected spring and rainwater collection [8]. The Indonesian definition further specifies that households are considered to have access to improved drinking water: (a) if the distance between the improved drinking water source and the wastewater disposal was less than 10 metres, but households used an improved water source for bathing/washing; or (b) if households used unimproved drinking water sources, including bottled water, refillable packaged drinking water, unprotected well, unprotected spring and river/stream, but used an improved water source for bathing/washing [17,18].

Access to improved sanitation facilities was defined as the proportion of households using an improved sanitation facility. Improved sanitation facilities are those designed to hygienically separate excreta from human contact, and the global MDG indicator considers toilets and protected latrines with wastewater disposal through sewer lines, septic tanks or on-site pits as improved sanitation facilities. MDG monitoring excluded facilities that are improved but are shared, while for SDG monitoring, shared facilities is considered as a limited sanitation service [8]. In the Indonesian context, improved sanitation facilities include flush toilets or pour flush toilets with wastewater disposal to wastewater treatment facilities or tanks with or without cement base/ground. Households are considered to have access to improved sanitation facilities if they use their own facilities or if they share with other households [18].

In total, 285,908 households that had complete data on access to improved drinking water and sanitation were included in the analysis. Data were disaggregated by province (n = 34) and district (n = 510), according to the geographical naming and boundaries of the Indonesian Ministry of Home Affairs [19]. The number of districts within provinces ranged from 5 to 38.

Disaggregated data were calculated using SPSS, taking into account the complex survey sampling design, including stratification, cluster sampling and sample weights. Disaggregated data were used to calculate summary measures of inequality to quantify the levels of inequality [20]. Within each province, the mean difference from mean (MDM) and the weighted index of disparity (IDIS – W) were calculated to measure absolute and relative inequality, respectively, between the districts in that province. These summary measures are appropriate to measure subnational regional health inequalities, especially with larger numbers of subnational regions; they have advantages over other types of summary measures (like variance and Theil index), including intuitiveness and the ability to calculate analogous absolute and relative measures [21]. MDM shows the average absolute difference between each district and the province average. It is calculated as the weighted sum of absolute differences between the district estimates ($y_{j}$) and the province average (μ):

(1) $$MDM=\underset{j}{\sum^{\;}}p_{j}\left|y_{j}-\mu \right|$$

IDIS – W is a relative measure of inequality that shows the average relative difference between each district and the province average. It is calculated as the weighted sum of absolute differences, divided by the province average:

(2) $$IDIS\;-W=\frac{{\sum^{\;}}_{j}p_{j}\left|y_{j}-\mu \right|}{\mu }*100$$

Both measures take into account the population size of each district by weighting differences by the district population share ($p_{j}$) of the province population. The Health Equity Assessment Toolkit Plus (HEAT Plus) was used to calculate these measures [22].

## Results

### Inequalities between provinces

Overall, more than two-thirds of households had access to improved drinking water (71.0%) and nearly two-thirds of households had access to improved sanitation (62.1%); performance varied between provinces (Table 1). Access to both improved drinking water and sanitation was highest in DKI Jakarta (93.4% and 89.3%, respectively). Access to improved drinking water was lowest in Bengkulu (41.1%) and Papua (51.3%), while access to improved sanitation was lowest in East Nusa Tenggara (23.9%) and Papua (28.0%).

**Table 1.** Access to improved drinking water and sanitation in Indonesia: national and provincial averages (SUSENAS, 2015).

Provinces, grouped by island/area	Access to improved drinking water (95% CI)	Access to improved sanitation (95% CI)	Number of districts
**National average**	71.0 (70.6–71.4)	62.1 (61.7–62.6)	510
**Sumatra**			
Aceh	61.2 (59.3–63.2)	54.7 (52.6–56.7)	23
North Sumatra	71.4 (70.1–72.8)	67.9 (66.5–69.2)	33
West Sumatra	66.6 (64.7–68.4)	45.0 (43.0–47.1)	19
Riau	74.2 (72.3–76.2)	51.3 (49.1–53.5)	12
Jambi	62.7 (60.3–65.2)	58.2 (55.7–60.7)	11
South Sumatra	65.2 (63.1–67.2)	61.3 (59.3–63.3)	17
Bengkulu	41.1 (38.2–44.0)	39.2 (36.0–42.6)	10
Lampung	55.1 (53.0–57.1)	44.8 (42.6–47.1)	15
Bangka-Belitung Islands	68.0 (65.0–71.0)	80.8 (78.4–83.0)	7
Riau Islands	84.1 (81.9–86.3)	72.0 (67.3–76.2)	7
**Java-Bali**			
DKI Jakarta	93.4 (92.3–94.5)	89.3 (86.8–91.3)	6
West Java	67.2 (65.9–68.5)	59.4 (58.0–60.9)	27
Central Java	73.6 (72.5–74.7)	67.2 (66.0–68.4)	35
DI Yogyakarta	81.0 (78.4–83.6)	86.3 (83.9–88.4)	5
East Java	76.6 (75.6–77.7)	63.5 (62.3–64.6)	38
Banten	67.7 (65.6–69.8)	67.0 (64.6–69.4)	8
Bali	91.3 (89.8–92.8)	85.5 (83.5–87.2)	9
**Nusa Tenggara Islands**			
West Nusa Tenggara	71.7 (69.1–74.3)	63.7 (61.0–66.4)	10
East Nusa Tenggara	62.7 (60.6–64.8)	23.9 (22.1–25.8)	22
**Kalimantan**			
West Kalimantan	68.4 (66.3–70.5)	39.8 (37.4–42.2)	14
Central Kalimantan	57.0 (54.1–59.9)	35.9 (33.4–38.5)	14
South Kalimantan	62.2 (59.9–64.6)	60.1 (57.8–62.4)	13
East Kalimantan	78.1 (75.2–81.1)	68.8 (65.2–72.2)	10
North Kalimantan	84.6 (81.2–88.0)	48.4 (43.6–53.3)	5
**Sulawesi**			
North Sulawesi	71.5 (69.2–73.9)	66.8 (64.1–69.4)	15
Central Sulawesi	61.5 (58.8–64.2)	55.4 (52.7–58.0)	13
South Sulawesi	72.1 (70.5–73.6)	72.4 (70.8–73.8)	24
Southeast Sulawesi	77.2 (74.9–79.5)	63.6 (61.1–66.1)	14
Gorontalo	66.5 (63.0–70.0)	55.0 (51.5–58.4)	6
West Sulawesi	53.9 (49.9–57.9)	51.2 (47.5–54.9)	6
**Maluku Islands**			
Maluku	65.0 (61.4–68.5)	60.0 (57.0–62.9)	11
North Maluku	60.1 (56.6–63.6)	59.2 (55.9–62.4)	10
**Papua**			
West Papua	68.9 (64.8–72.9)	62.8 (58.6–66.8)	13
Papua	51.3 (48.9–53.7)	28.0 (26.1–30.0)	28

**Figure 1.**
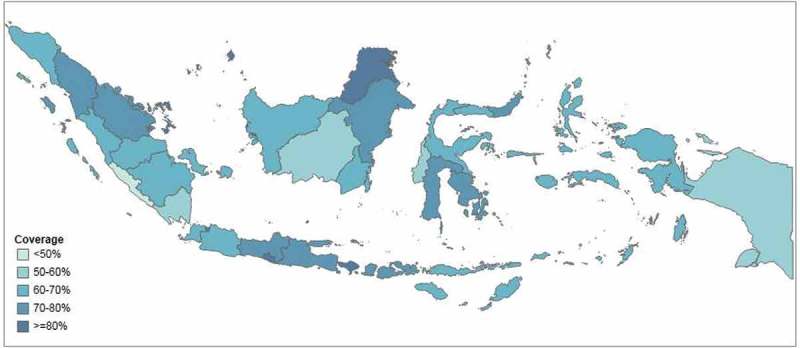
Access to improved drinking water in 34 provinces in Indonesia (SUSENAS 2015).

**Figure 2.**
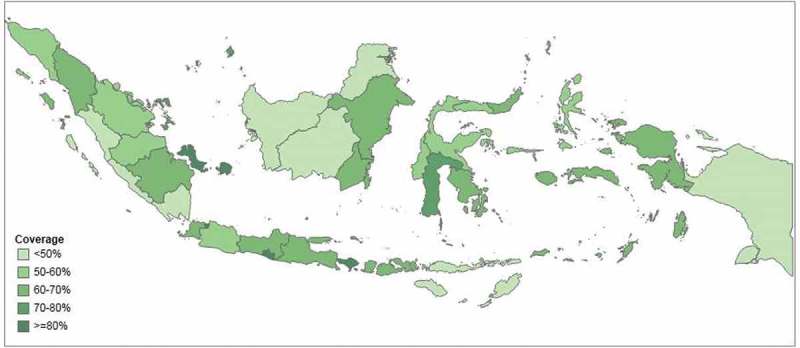
Access to improved sanitation in 34 provinces in Indonesia (SUSENAS 2015).

Figures 1 and 2 illustrate the levels of coverage across provinces. In five provinces, more than 80% of households had access to improved drinking water (Bali, DI Yogyakarta, DKI Jakarta, North Kalimantan and Riau Islands) and in four provinces, more than 80% of households had access to improved sanitation (Bali, Bangka-Belitung Islands, DI Yogyakarta and DKI Jakarta). In one province, (Bengkulu) less than 50% of households had access to improved drinking water, and access to improved sanitation was less than 50% in eight provinces (Bengkulu, Central Kalimantan, East Nusa Tenggara, Lampung, North Kalimantan, Papua, West Kalimantan and West Sumatra).

### Inequalities within provinces

**Figure 3.**
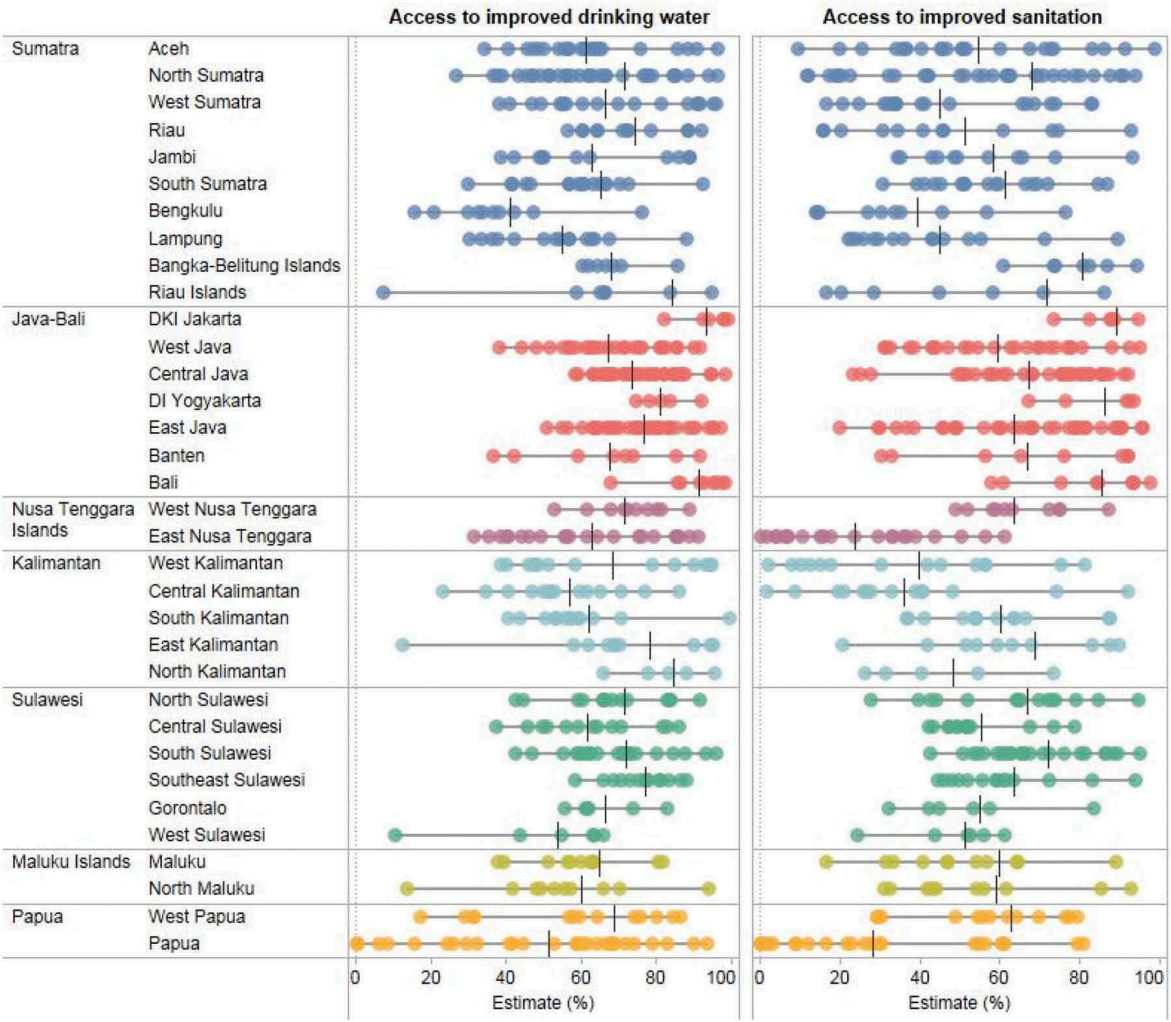
Access to improved drinking water and sanitation in 510 districts across 34 provinces in Indonesia (SUSENAS 2015).

Access to improved drinking water and sanitation also varied between districts within each province (Figure 3). In some cases, the variations were substantial. For instance, in Riau Islands, 9 out of 10 households in Batam district had access to improved drinking water, while only 1 out of 10 households in Kepulauan Anambas district reported access. Similarly, in East Kalimantan, 9 out of 10 household in Balikpapan, Bontang and Samarinda districts had access to improved drinking water compared with only 1 out of 10 households in Mahakam Hulu district. In Papua, a large variation between districts could be observed for both access to improved drinking water and sanitation. While Papua was among the provinces with the lowest access, 9 out of 10 households in Jayapura and Mimika districts and 8 out of 10 households in Biak Numfur and Jayapura districts had access to improved drinking water and sanitation, respectively; conversely, almost no households had access to improved drinking water and sanitation in Mamberamo Tengah and Lanny Jaya districts.

**Figure 4.**
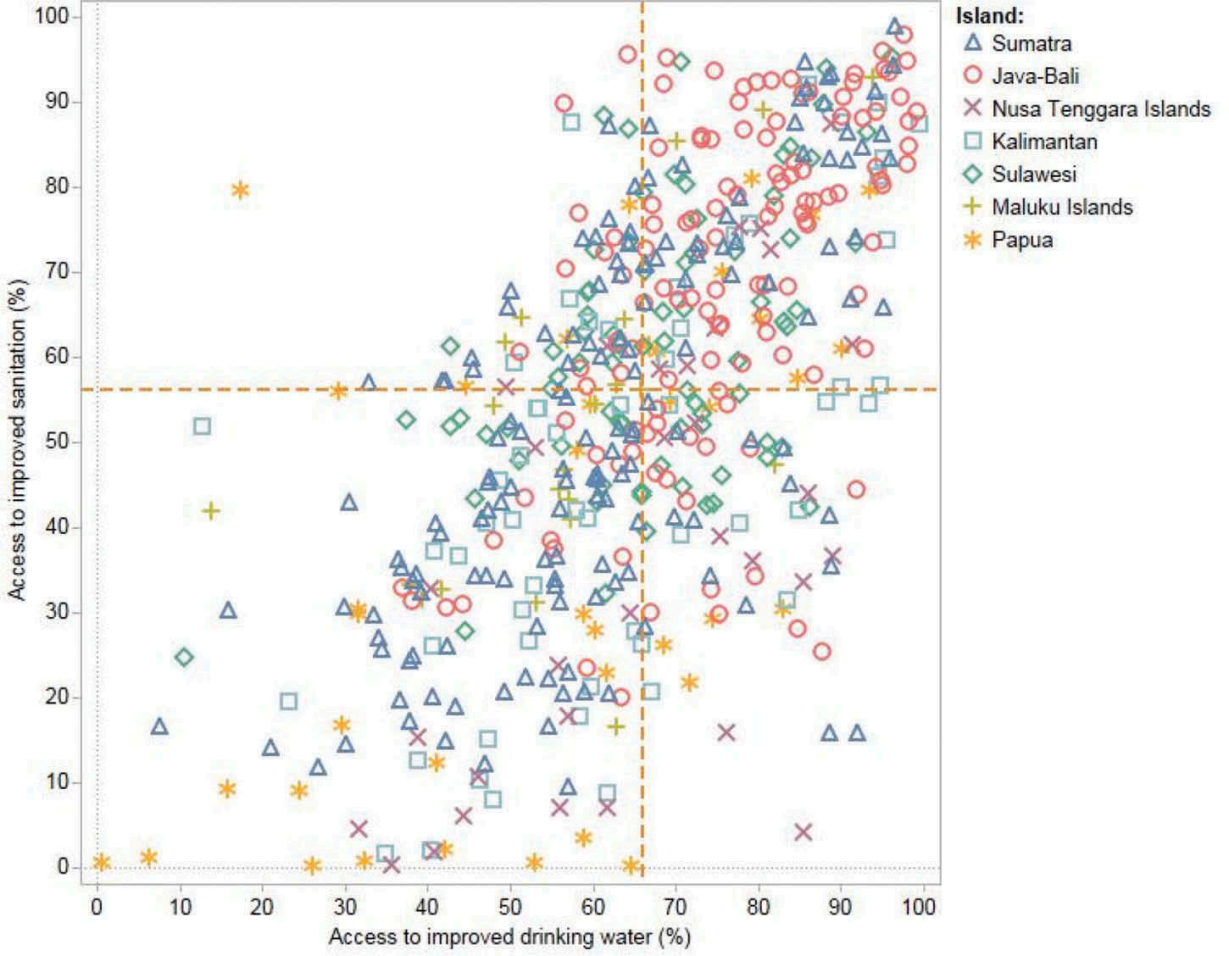
Access to improved drinking water and sanitation in 510 districts across 7 islands/areas in Indonesia (SUSENAS 2015).

Across the 510 districts, access to improved sanitation was positively associated with the level of access to improved drinking water (correlation coefficient = 0.63) (Figure 4). In most districts, access to improved drinking water was higher than access to improved sanitation, although there were exceptions. A clustering pattern was observed by island. For example, districts in Java-Bali reported the highest levels of access to improved drinking water and sanitation (median of 75.4% and 72.7%, respectively), while districts in Maluku Islands presented the lowest levels of access to improved drinking water (median = 57.2%), and districts in Papua presented the lowest levels of access to improved sanitation (median = 30.3%).

Table 2 presents the absolute and relative inequality in access to improved drinking water and sanitation across districts within each province. Provinces with the same (or similar) numbers of districts showed different levels of absolute and/or relative inequality. For example, in Maluku and North Maluku, an average of about 60% of households had access to improved drinking water, but within-province inequality was greater in North Maluku compared with Maluku. In North Maluku, each district had, on average, a 15.0 percentage point difference in access compared with the province average, while this difference was 10.0 percentage points in Maluku. Relative inequality was 1.6 times greater in North Makulu than in Makulu (IDIS – W = 25.0 vs. IDIS – W = 15.4, respectively). Similarly, in South Kalimantan and West Kalimantan, more than 60% of households had access to improved drinking water, but both absolute and relative within-province inequality were larger in West Kalimantan than South Kalimantan (MDM = 21.4 percentage points vs. MDM = 14.0 percentage points and IDIS – W = 31.4 vs. IDIS – W = 22.5, respectively).

**Table 2.** Access to improved drinking water and sanitation: absolute and relative within-province inequality in 34 Indonesian provinces (SUSENAS 2015).

	Access to improved drinking water		Access to improved sanitation		
Provinces, grouped by island/area	Absolute inequality* (percentage points)	Relative inequality**	Absolute inequality* (percentage points)	Relative inequality**	Number of districts
**Sumatra**					
Aceh	11.4	18.7	16.8	30.7	23
North Sumatra	15.1	21.1	19.4	28.5	33
West Sumatra	14.0	21.1	15.9	35.4	19
Riau	10.5	14.1	23.2	45.2	12
Jambi	16.6	26.4	16.5	28.3	11
South Sumatra	12.6	19.4	13.4	21.8	17
Bengkulu	13.7	33.3	17.2	43.8	10
Lampung	11.1	20.1	16.0	35.7	15
Bangka-Belitung Islands	6.0	8.8	7.5	9.2	7
Riau Islands	13.3	15.8	17.6	24.5	7
**Java-Bali**					
DKI Jakarta	5.1	5.5	2.7	3.1	6
West Java	12.1	18.1	16.3	27.4	27
Central Java	7.6	10.3	13.4	19.9	35
DI Yogyakarta	4.9	6.1	9.0	10.5	5
East Java	8.8	11.4	16.2	25.5	38
Banten	14.1	20.8	18.4	27.5	8
Bali	5.9	6.5	11.5	13.4	9
**Nusa Tenggara Islands**					
West Nusa Tenggara	7.0	9.8	7.0	11.0	10
East Nusa Tenggara	15.7	25.1	16.1	67.4	22
**Kalimantan**					
West Kalimantan	21.4	31.4	22.9	57.6	14
Central Kalimantan	13.6	23.8	22.5	62.8	14
South Kalimantan	14.0	22.5	14.4	24.0	13
East Kalimantan	13.3	17.0	16.2	23.5	10
North Kalimantan	7.9	9.4	18.3	37.8	5
**Sulawesi**					
North Sulawesi	8.9	12.5	11.3	16.8	15
Central Sulawesi	12.0	19.6	10.0	18.0	13
South Sulawesi	10.6	14.7	13.0	17.9	24
Southeast Sulawesi	6.0	7.8	11.0	17.3	14
Gorontalo	8.0	12.1	11.4	20.7	6
West Sulawesi	13.1	24.4	8.7	16.9	6
**Maluku Islands**					
Maluku	10.0	15.4	17.0	28.4	11
North Maluku	15.0	25.0	18.1	30.6	10
**Papua**					
West Papua	15.2	22.1	14.5	23.2	13
Papua	25.5	49.7	25.9	92.3	28

*Absolute inequality is measured by the Mean Difference from Mean (MDM).

**Relative inequality is measured by the Weighted Index of Disparity (IDIS – W)

Similar patterns were evident for improved sanitation. More than 50% of households had access to improved sanitation in Gorontalo and West Sulawesi, yet there were greater levels of absolute and relative within-province inequality in Gorontalo compared with West Sulawesi (MDM = 11.4 percentage points vs. MDM = 8.7 percentage points and IDIS – W = 20.7 vs. IDIS – W = 16.9, respectively). In Central Java and East Java, more than 6 out of 10 households had access to improved sanitation, but within-province inequality was larger in East Java than Central Java. In East Java, access in each district varied, on average, by about 16.2 percentage points from the province average, while in Central Java the variation was on average about 13.2 percentage points. Relative inequality was 1.3 times higher in East Java than Central Java (IDIS – W = 25.5 vs. IDIS – W = 19.9, respectively).

**Figure 5.**
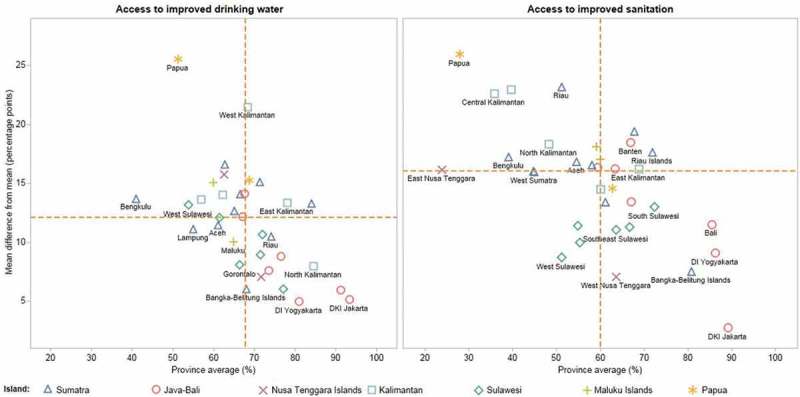
Access to improved drinking water and sanitation: province average and absolute within-province inequality in 34 provinces across 7 islands/areas in Indonesia (SUSENAS 2015).

Considering the provincial average alongside absolute within-province inequality for access to improved drinking water and access to improved sanitation (Figure 5), there were both differences and similarities between the two indicators. Overall, we found greater coverage and lower absolute within-province inequality for access to improved drinking water than for access to improved sanitation. Papua was the province with the highest levels of absolute within-province inequality in both access to improved drinking water and sanitation.

## Discussion

This study demonstrated that, while the majority of Indonesian households had access to improved drinking water and sanitation, inequalities between and within provinces were pervasive. We found a strong association between the district where people live, and the level of access to improved drinking water and sanitation. These results are indicative of uneven development across Indonesia, which was influenced by the political process of decentralization of public services and administration that occurred in the 1980s and 1990s [23].

Households in the provinces of Java-Bali islands tended to have the greatest access to improved drinking water and sanitation, and these provinces also reported the lowest within-province inequality. This was unsurprising, as Java-Bali has a strong economy, and also performs well across other health topics [23,24]. Conversely, the provinces of Papua and Sumatra tended to report the lowest access to improved drinking water and sanitation, and had the highest within-province inequality. In certain districts, households reported alarmingly low levels of coverage. For instance, in the Kepulauan Anambas district of Riau Islands, and the Dogiyai, Mamberamo Tengah, Lanny Jaya and Tolikara districts of Papua, household access to improved drinking water was around 10% or less. Access to improved sanitation was around 5% or less in the Lembata, Manggarai Timur, Sumba Barat Dara and Sumba Tengah districts of Nusa Tenggara Islands, Sekadau district of West Kalimantan and Kapuas district of Central Kalimantan, and Asmat, Deiyai, Lanny Jaya, Paniai, Puncak, Tolikara, Yalimo, Yahukimo districts of Papua. Previous research has suggested that bottlenecks in water and sanitation service delivery may be linked to issues surrounding planning and budgeting, as well as shortcomings in the output and uptake of services [25]. Capacity-building efforts in poor-performing provinces and districts may be warranted to promote and support effective implementation of policies and strategies, including equity-oriented infrastructure development and resource allocation.

Our results corroborate the practical importance of within-country inequality monitoring by subnational regions [26]. Namely, this approach to monitoring by geographical area leads to an intuitive understanding of health inequalities and can serve to identify practical avenues for intervention: disadvantaged subgroups are easy to identify and locate. Further, the results of monitoring by subnational regions can be used for benchmarking, and to guide resource allocation, planning and evaluation efforts. Monitoring by subnational regions is particularly relevant for the topic of improved drinking water and sanitation in Indonesia, as many major initiatives addressing this topic have been implemented and/or administered at local levels. For example, the Community-Led Total Sanitation Program, which targets rural communities to encourage the use of improved sanitation facilities [27,28], has been integrated as part of the national strategy to address universal coverage of safe water and sanitation [29]. The Water & Sanitation for Low Income Communities Project works at the community level to develop action plans for integrated water supply, sanitation and hygiene improvement [30]. The district-level and provincial governments are important players in promoting the success of community initiatives through supporting enabling environments, demand creation and supply improvements. Monitoring and reporting inequalities serves as an evidence basis to guide interventions that strengthen the capabilities of poor-performing districts and provinces. The SUSENAS has been conducted since 1979, so future studies can be done to assess subnational performance and inequality in water and sanitation over the past 30 years (and thus situate our results within historical trends).

Our study benefited from the use a large set of population-representative data collected at the household level, which allowed us to make comparisons of indicators across districts. The indicator definitions adopted for this study reflect national considerations for assessing improved drinking water and sanitation in Indonesia, as developed by the National Development Planning Agency/BAPPENAS. Thus, the definition applied here provides a more relevant measure for national circumstances than standardized global definitions [8], which rely on a narrower, consistent set of technical classifications that may be applied across multiple country settings. The global definitions of indicators of drinking water, sanitation and hygiene have been revised to meet the ambition of the new SDG targets which emphasize the safe management of drinking water and sanitation facilities. The concept of equitable access for all is addressed through disaggregation of results; for example, by wealth and place of residence (urban/rural). Additionally, the 2030 Agenda for Sustainable Development calls for prioritizing global monitoring in institutional settings such as schools and health care facilities as well as in households [8]. While Indonesia’s data collection system including the SUSENAS was well aligned with the MDG indicators of improved water and sanitation, there are data gaps regarding some of the new elements of the SDG indicator definitions of safely managed drinking water and sanitation services. Data on drinking water quality and management of excreta from on-site sanitation systems, for example, are not yet available for calculation of SDG baselines. It is likely that the inequalities in access to good-quality drinking water, and well-managed sanitation systems, are at least as great as those observed in access to improved water and sanitation. As data gaps about the quality of services are filled, it remains important to ensure that data are available from all regions of the country, and that inequality measures are applied to the higher-level indicators as well as the basic ones.

The interpretation of summary measures of inequality across provinces with different numbers of districts requires some caution. A resolution problem emerges when inequality measures are calculated using variable numbers of subunits (i.e. districts) [21]. As a result, provinces with larger numbers of districts will tend to show larger levels of inequality than provinces with fewer districts. Comparisons across provinces with the same or similar number of districts limits the resolution problem, which is inherent in any summary measure of regional inequality.

## Conclusion

In conclusion, the findings of this study demonstrate that access to improved drinking water and sanitation is a pressing concern in certain subnational regions of Indonesia. To our knowledge, this is the most recent analysis of access to improved drinking water and sanitation in Indonesia. Regular monitoring of within-country subnational inequality in Indonesia is key to track progress towards global and national commitments that aim to ensure universal access to safe and affordable drinking water, and adequate and equitable sanitation and hygiene for all. Monitoring inequalities across subnational regions can provide an important evidence base to help inform equity-oriented efforts to achieve accelerated gains in poorer-performing areas.

## Paper context

In Indonesia, lack of access to improved drinking water and sanitation is a prominent public health issue; reporting in this area has tended to focus on national data, with a lack of information about subnational inequalities. In this paper, we report our analyses of within-country regional inequality in household access to improved drinking water and sanitation. Our results serve as a key benchmark for tracking progress towards global and national commitments that promote universal access to safe and affordable drinking water, and adequate and equitable sanitation and hygiene for all. Monitoring and reporting health inequalities across subnational regions can provide an evidence basis to inform equity-oriented efforts to achieve accelerated gains in poorer-performing areas.
